# Prevalence of human immunodeficiency virus-1 drug-resistant mutations among adults on first- and second-line antiretroviral therapy in a resource-limited health facility in Busia County, Kenya

**DOI:** 10.11604/pamj.2020.37.311.25909

**Published:** 2020-12-03

**Authors:** Olipher Makwaga, Maureen Adhiambo, David Hughes Mulama, John Muoma, Ferdinard Adungo, Humphrey Wanjiku, Asiko Ongaya, Geoffrey Mutisya Maitha, Matilu Mwau

**Affiliations:** 1 Kenya Medical Research Institute, Busia, Kenya,; 2 Masinde Muliro University of Science and Technology, Kakamega, Kenya,; 3 East African Health Research Commission, Nairobi, Kenya,; 4 AIDS Healthcare Foundation, Nairobi, Kenya

**Keywords:** HIV-1, antiretroviral therapy, drug-resistance, mutations

## Abstract

**Introduction:**

in Kenya, about 1.5 million people are living with the Human Immunodeficiency Virus (HIV). Antiretroviral therapy aids in viral suppression. However, drug-resistance threaten the gains of the HIV infection control program. To determine the prevalence of HIV-1 drug-resistant mutations among adults on ARV therapy attending Khunyangu sub-county hospital in Busia County, Kenya, 50 blood samples were analyzed.

**Methods:**

the samples were collected from November 2019 to January 2020 and tested for HIV-1 viral load. HIV-1 drug-resistance was analyzed through the sequencing of the HIV-1 pol gene. Generated sequences were aligned using RECall (beta v3.05) software. HIV-1 drug-resistance was determined using the Stanford University HIV database.

**Results:**

females were 34 and males 16. The general prevalence of HIV-1 drug-resistance was 68%. Out of 34 participants on first-line drugs, 59.9% had mutations against these drugs and 5.9% against the second-line drugs. Out of 16 participants on second-line drugs, 43.8% had mutations against these drugs and 50% against the first-line drugs. The prevalence of mutations encoding resistance to Nucleotide reverse transcriptase inhibitors (NRTIs) were 23(46%); Non-nucleotide Reverse transcriptase inhibitors (NNRTIs), 29(58%) and protease inhibitors (PIs), 7(14%). Dual and multi-class HIV-1 drug-resistance prevalence was as follows: NRTIs + NNRTIs 16(32%); NRTIs + NNRTs + PIs 4(8%); NRTIs + PIs 1(2%). A total of 126 mutations were identified. Predominant NNRTIs mutations were K103N (15), Y181C (9), G190A (7), and H221Y (6) NRTIs, M184V (17), Y115F (5) and PIs, I54V (4).

**Conclusion:**

the study demonstrates a high prevalence of HIV-1 drug-resistance which calls for intervention for the strengthening of health programs.

## Introduction

Globally, 38 million persons are living with the Human Immunodeficiency Virus (HIV). About 1.7 million people were newly infected and 690,000 HIV related death occurred in 2019. The problem of the epidemic varies regionally. Sub-Saharan Africa bares the greatest burden with one out of 25 adults living with HIV [1]. Kenya ranks 12^th^ with the highest HIV-1 epidemic worldwide with 1.5 million people living with the virus (1,388,200 of age 15 years and above and 105,200 children 0 to 14 years) [2]. HIV-1 prevalence among adults´ in the country stands at 4.9% with 52,800 newly infected cases across all ages annually [3]. Approximately 28,200 deaths related cases were observed in 2018 [2].

In Kenya, Busia is ranked fifth with the highest HIV-1 prevalence of 38606 (7.7%) [2]. ARV therapy is recommended for all people infected with HIV-1 in Kenya because it reduces the HIV-1 viral load while improving their immunity against other infections [4]. About 1,035,615(75%) infected adults and 86323(84%) children are receiving ARV therapy country-wide. Subsequently, 33654 (95%) adults and 3078 (78%) children are receiving antiretroviral therapy in Busia County [2]. This is because many newly infected people tested HIV-1 positive are started on ARVs immediately without considering CD4 levels. Furthermore, AMPATH as a None Governmental Organization (NGO) is playing a major role of ensuring HIV-1 drugs are available to patients, new patients are enrolled for ARVs and old ones are retained on ARVs by providing food to HIV-1 infected individuals and their dependent in Busia county [5]. Regardless of these milestones in the treatment of HIV, HIV-1 drug-resistance remains a critical issue due to the development of mutations that helps the virus to evade and multiply in the presence of active drugs thus threatening the victory of treatment regimens.

Three groups of ARVs are available in the treatment of HIV-1 in Kenya. These are NRTIs namely, Abacavir (ABC), Lamivudine (3TC), Tenofovir disoproxil fumarate (TDF), Azidovodine (AZT), Dolutegravir (DTG); NNRTIs namely, Efavirenz (EFV), Nevirapine (NVP) and PIs are Atazanavir/ritonavir (ATV/r) and Lopinavir/ritonavir (LPV/r). Initially, some of these drugs were used as a single dose in the treatment of HIV-1 infections. The development of HIV-1 drug-resistance triggered the combination of these drugs to meet the treatment threshold. These combinations include: one NRTI with Lamivudine (NRTI) as a compulsory drug and one NNRTI and are grouped as first-line therapy. One NRTI with Lamivudine as compulsory drug and one Protease (PI) and are grouped as second-line therapy. However, the combination regimens are also threatened by the development of mutations by the virus against them. Past studies have identified 13.8% among Kenyan injecting drug users, while another study identified a drug mutation prevalence of 23.1% among general adult population [6, 7]. However, these studies were carried out prior to the World Health Organization guidelines that everyone infected with HIV to be initiated to drugs regardless of CD4 tally. Therefore, the current study aimed at establishing HIV-1 drug-resistance after the guidelines [8]. Much as ARVs are important in viral suppression, mutations encoded by the virus against specific classes of ARVs may render them ineffective.

Past studies in Kenya have identified mutations conferring resistance to specific classes of ARVs [9-11]. At the coastal region of Kenya, L90M, M46I and D30N mutations conferring resistance to PIs were identified among out-patients injecting drug users [9]. A study on HIV-1 drug-resistance patterns in Nairobi identified M184V, K65R, T215Y and K70R mutations conferring resistance to NRTIs and K103N, G190A, V106A, Y184V, A98G, Y181C mutations conferring resistance to NNRTIs [10]. The past published study on HIV-1 drug-resistance in Busia County in which the prevalence of HIV-1 drug-resistance was 22.6% focused on children aged between 6 weeks and 5 years in which some mutations conferring resistance to NNRTIs and NRTIs were identified [11]. However, current data about resistance in individual who are older than the stated above in Busia county is missing. Therefore, this study was designed to look at the current HIV-1 drug-resistance in adults. In Kenya, many studies on the HIV-1 drug-resistances were carried out in urban centers [12-15], however, reports on the general HIV-1 drug-resistance is missing especially in resource-limited rural settings with a longer history of ARV use. Therefore, this study was aimed at determining the prevalence of HIV-1 drug-resistant mutations among adults on first- and second-line antiretroviral therapy in a resource-limited health facility in Busia County, Kenya. The findings from this study are important to people living with HIV in rural settings, which comprise the majority of those affected with HIV.

## Methods

**Study design, study site and population:** this was a cross sectional study involving adult participants of Busia County attending a Comprehensive Care Clinic of Khunyangu sub-county referral hospital from November 2019 to January 2020. Busia county is one of the 47 Kenyan counties. Busia county ranks fifth with the highest HIV-1 prevalence in the country. Furthermore, its prevalence is more than the national [2]. It is located along the Kenya-Uganda border. Khunyangu sub-county referral hospital where the study took place, is a reference level four hospital in the county with a high volume hospital patient attendance. The hospital is also among the health facilities which benefits from AMPATH Integrating Nutrition Support initiative in Busia county where newly HIV infected persons are actively enrolled for ARVs and old ones are retained on ARVs by providing food to HIV infected individuals and their dependents [5]. HIV-1 infected participants, on ARVs treatment aged ≥18 years that gave consent to participate were recruited.

**Sample size determination:** sample size formula by [16] was used to determine the minimum number of samples:

$$\frac{{\text{N=4(crit)}}^{\text{2}}\text{p(1-p)}}{{\text{D}}^{\text{2}}}$$

N= minimum sample size; Zcrit is the standard normal deviation= 1.96 (standard errors from the mean) at D= 0.05% level of significance, p= prevalence of infection. The prevalence of 1.1% of HIV Type 1 drug-resistance among adults in a rural HIV clinic in Kenya [15] being too low for this study, we averaged these by an overall prevalence of 4.7% [17] of drug-resistant HIV-1 transmission in Africa to compute our sample size and this was 2.9%. p= 0.029 HIV-1 drug resistance prevalence, therefore, n= 43 blood samples which were rounded to 50.

**Sampling procedure:** all adult patient attending a comprehensive care Clinic of Khunyangu sub-county referral hospital, already started on ART and consented to participate in the study were sampled systematically. Filling a simple questionnaire on demographics (age and sex) and duration on ARVs were done. Approximately 5 ml of blood samples were obtained using sterile needles and syringes in EDTA tubes by qualified medical laboratory Technologists. Triple packaging of the samples was done and transported to Alupe KEMRI laboratory following the national sample shipment program for clinical specimens. Samples were removed from the cold environment and allowed to come to room temperature for at least one hour before processing.

**Viral load and HIV-1 drug-resistance testing:** samples for viral load testing were collected in plasma preparation tubes (Becton Dickinson, San Jose, CA, USA). The plasma was aliquoted into cryotubes. RNA extraction and quantification was done using abbott sample processing/abbott real time machine (abbott molecular Inc. USA) and cobas ampliprep/cobas taqman HIV-1 test v.2.0 (roche diagnostic, USA) according to manufacturer´s instructions [18, 19].

RNA was transcribed into cDNA using an in-house reverse transcriptase polymerase chain reaction (RT-PCR) protocol using thermoFisher scientific´s genotyping kit. The extracted RNA was denatured at 65°C for 10 minutes. The RT- PCR was done under the following cycling conditions: One cycle of reverse transcription at 50°C for 45 minutes, one cycle of enzyme inactivation at 94°C for 2 minutes, 40 cycles of denaturation at 94°C for 15 seconds, 40 cycles of annealing at 50°C for 20 seconds, 40 cycles of extension at 72°C for 2 minutes and one cycle of final extension at 72°C for 10 minutes. The nested PCR was done under the following cycling conditions: one cycle of initial denaturation at 94°C for 4 minutes, 40 cycles of denaturation at 94°C for 15 seconds, 40 cycles of annealing at 55°C for 20 seconds, 40 cycles of extension at 72°C for 2 minutes and one cycle of final extension at 72°C for 10 minutes. Agarose gel (1%) was used to confirm the PCR amplified results. Gel electrophoresis was done for confirmation of the amplified DNA bythe gel visualization on the imaging system and photographed. Purification of the PCR products was performed using Thermo Fisher Scientific´s Clean Sweep Purification Reagent under the following conditions: Digest at 37°C for 15 minutes and heat deactivation at 80°C for 15 minutes. Cycle sequencing was done using 6 sequencing mixes (F1, F2, F3, R1, R2, and R3) and PGEM sequencing control. The cycle sequencing conditions was set at 25 cycles of denaturation, annealing and extension at 96°C for 10 seconds, 50°C for 5 seconds, and 60°C for 4 minutes respectively. Purification of cycle sequencing products was done using thermo fisher scientific´s bigdye X-terminator purification Kit.

Direct sequencing of the pol gene encoding protease (codons 6-99) and reverse transcriptase (codons 1-239) was done on the 3730XL DNA analyzer (applied biosytems) using sanger sequencer. Base calling was facilitated by seqscanner v.6 (applied biosystems Inc. USA). This was by evaluating the main technical parameters (i.e., raw data, electropherograms and the quality value of sequenced bases). RECall (beta v3.05) software was used for aligning and generating the consensus sequences. And drug-resistance was determined using the International Aids Society (IAS) algorithm and the Stanford University HIV database.

**Ethical approval:** all ethical procedures were considered. This study was approved by ethical review unit of Kenya Medical Research Institute number study KEMRI/SERU/CIPDCR/008/3333.

**Statistical analysis:** data analysis were conducted using SPSS version 20 (Armonk, NY: IBM Corp). Descriptive statistics were done to analyze socio-demographic factors and the frequency of various HIV-1 drug-resistant mutations. Chi-square and fisher´s exact tests were used to analyze the associations of two variables (duration of ARV uptake and mutations, mutations in females and males, mutations and participants on first versus second-line therapy). All tests were two tailed, and p values <0.05 were considered significant.

## Results

### Patients characteristics

In February 2020, a total of 50 (female, 68.0% and male, 32.0%) participants were considered for this study. Their age was between 18 to 59 years (mean, 29.3 and median, 24.5 years). In general, participants were taking 7 different types of ARVs with the majority taking first-line ARVs therapy compared to the second-line. Many patients were taking AZT+3TC+NVP, TDF+3TC+EFV and AZT+3TC+EFV ARVs regimen combination (Table 1).

**Table 1:** mutations, viral load, treatment regimen

Sample code	Accession no. in gene bank	Viral load (copies/ml)	Age	Gender	Treatment regimen	Line of ARVs	NRTIs mutations identified	NNRTIs mutations identified	PIs mutations identified
001K	MT723997	90880	20	M	AZT+3TC+ATV/r	second	D67N,M184V,T215F, K219E	G190A	G48V,I54V,V82A
2K	MT853184	46907	42	F	AZT+3TC+NVP	first	None	K103N	None
3K	MT853185	89000	59	F	TDF+3TC+ATV/r	second	A62V,V65R	A98G,K101E,Y181C, G190A	None
4K	MT853186	123502	18	F	AZT+3TC+NVP	first	M184V	None	None
05K	MT853187	54090	41	M	TDF+3TC+DTG	first	K70E,M184I	K101E,Y181C	None
06K	MT853188	262080	20	F	AZT+3TC+ATV/r	second	L74I,M184V	A98G,K103N,P225H	None
07K	MT853189	4950	21	M	AZT+3TC+ATV/r	second	V75I,M184V	Y181C,H221Y	None
08K	MT853190	260186	18	F	AZT+3TC+EFV	first	L74V,M184V,Y115F	K103N,V179E,Y181C	None
09K	MT853191	26289	20	F	TDF+3TC+EFV	first	None	K103N	None
10K	MT853192	10593	18	M	AZT+3TC+NVP	first	M184V	K103N	None
11K	MT853193	2683	36	F	AZT+3TC+NVP	first	None	K103S	None
12K	MT853194	39630	27	F	AZT+3TC+ATV/r	second	D67N,M184V,K70E	K101E,E138A,G190S	K20T
13K	MT853195	7732	45	M	AZT+3TC+EFV	first	None	K103N	None
14K	MT853196	147908	23	F	TDF+3TC+EFV	first	None	None	F53L
15K	MT853197	23316	33	F	TDF+3TC+EFV	first	D67N,K70T,T69D	None	None
16K	MT853198	18575	46	F	TDF+3TC+ATV/r	second	K70E	K103N,P225H	None
17K	MT853199	57886	23	F	TDF+3TC+ATV/r	second	M184V	K103N,E138A,G190A	None
18K	MT853200	46197	18	F	AZT+3TC+NVP	first	M184V	Y181C,H221Y	None
19K	MT853201	5026	24	F	TDF+3TC+EFV	first	K65R,M184V	K103N,M230L	None
20K	MT853202	20680	43	F	AZT+3TC+EFV	first	None	K101E,G190A,V179D	None
21K	MT853203	13234	49	F	TDF+3TC+EFV	first	K65R,Y115F	K103N,Y181C,V179F, H221Y	None
22K	MT853204	6964	23	M	AZT+3TC+NVP	first	None	Y181C,H221Y	None
23K	MT853205	416926	19	M	AZT+3TC+ATV/r	second	M84V	Y181I	None
24K	MT853206	147146	31	F	TDF+3TC+EFV	first	None	K101E	None
25K	MT853207	28506	53	F	AZT+3TC+EFV	first	M184V	K103N,V108I,M230L	None
26K	MT853208	66502	20	M	AZT+3TC+EFV	first	K65R,Y115F,M184V	V106I,Y188L	None
27K	MT853209	193920	20	F	AZT+3TC+NVP	first	None	G190A	None
28K	MT853210	15263	43	F	AZT+3TC+LPV/r	second	M184V	K101H,G190A,P225H	I54V,V82A,L10F
29K	MT853211	57819	27	F	AZT+3TC+V/r	second	None	K103N	None
30K	MT853212	11351	36	F	TDF+3TC+ATV/r	second	M184V,T215Y,M41L	None	G48A,I54V,V82S,Q58E
31K	MT853213	94730	21	F	TDF+3TC+EFV	first	None	None	I54V
32K	MT853214	5703	34	F	AZT+3TC+ATV/r	second	M184V,T215F	K103N,E138A	M46I,F53L,L89T
33K	MT853215	53359	41	F	AZT+3TC+ATV/r	second	K65R,Y115F	K103N,H221Y,V179F, Y181C	None
34K	MT853216	10656	18	F	AZT+3TC+EFV	first	L74V,M184V,Y115F	K103N,G190A	None
35K	MT957067	9000	30	F	TDF+3TC+EFV	first	None	None	None
36K	MT957068	2653	18	M	AZT+3TC+EFV	first	None	None	None
37K	MT957069	4849	18	F	AZT+3TC+NVP	first	None	None	None
38K	MT957070	1878	18	F	AZT+3TC+EFV	first	None	None	None
39K	MT957071	12589	18	M	AZT+3TC+NVP	first	None	None	None
40K	MT957072	714620	38	F	TDF+3TC+EFV	first	None	None	None
41K	MT957073	51737	47	M	AZT+3TC+NVP	first	None	None	None
42K	MT957074	54688	33	M	AZT+3TC+NVP	first	None	None	None
43K	MT957075	2959	25	F	TDF+3TC+DTG	first	None	None	None
44K	MT957076	2704	18	M	TDF+3TC+DTG	first	None	None	None
45K	MT957077	147908	23	F	AZT+3TC+LPV/r	second	None	None	None
46K	MT957078	714628	38	F	AZT+3TC+LPV/r	second	None	None	None
47K	MT957079	51737	47	M	AZT+3TC+NVP	first	None	None	None
48K	MT957080	2704	18	M	TDF+3TC+DTG	first	None	None	None
49K	MT957081	52323	18	M	AZT+3TC+NVP	first	None	None	None
50K	MT957082	62609	28	F	AZT+3TC+LPV/r	second	None	None	None

n: number of participants equivalent to sample size; ARVs: Antiretroviral; 3TC: Lamivudine; EFV: Efevirenz; LPV/r: Lopinavir/ritonavir; AZT: Azidovodine; ATV/r: Atazanavir/ ritonavir; NVP: Nevirapine; TDF: Tenofovir disoproxil fumarate; DTG: Dolutegravir; NRTIs: nucleotide reverse transcriptase inhibitors, NNRTIs: non-nucleotide reverse transcriptase inhibitors; PIs: Protease Inhibitors; SD: Stanford database.

The overall prevalence of HIV-1 drug-resistance of any kind was 68%. 59.9% participants on the first-line ARVs had mutations against these first line drugs and 5.9% had already mutations against the second-line drugs. 43.8% participants on the second-line ARVs had HIV-1 drug-resistant mutations to these second line ARVs and 50.0% participants on the second line had mutations against the first-line only but not against the second-line. All participants had a viral load of 100°Cp/ml and above (Table 1).

A total of 126 mutations were identified of which the majority were encoding resistance to the NNRTIs class of ARVs. Predominant NNRTIs mutations were K103N, Y181C, G190A, H221Y, and K101E; NRTIs were M184V, Y115F, K65R, K70R, D67N and PIs were I54V, F53L and V82A (Table 2).

**Table 2:** frequency of mutations identified in all the 50 participants

NNRTIs mutations, n: 64			NRTIs mutations n: 46			PIs mutations n: 16		
	Frequency	Percent		Frequency	Percent		Frequency	Percent
A98G	2	3.1	A62V	1	2.2	F53L	2	12.5
E138A	3	4.7	D67N	3	6.5	G48A	1	6.3
G190A	7	10.9	K219E	1	2.2	G48V	1	6.3
G190S	1	1.6	K65R	4	8.7	I54V	4	25.0
H221Y	6	9.4	K70E	3	6.5	K20T	1	6.3
K101E	5	7.8	K70T	1	2.2	L10F	1	6.3
K101H	1	1.6	L74I	1	2.2	L89T	1	6.3
K103N	15	23.4	L74V	2	4.3	M46I	1	6.3
K103S	1	1.6	M184I	1	2.2	Q58E	1	6.3
M230L	2	3.1	M184V	16	34.8	V82A	2	12.5
P225H	3	4.7	M41L	1	2.2	V82S	1	6.3
V106I	1	1.6	M84V	1	2.2	-	-	-
V108I	1	1.6	T215F	2	4.3	-	-	-
V179D	1	1.6	T215Y	1	2.2	-	-	-
V179E	1	1.6	T69D	1	2.2	-	-	-
V179F	3	4.7	V65R	1	2.2	-	-	-
Y181C	9	14.1	V75I	1	2.2	-	-	-
Y181I	1	1.6	Y115F	5	10.9	-	-	-
Y188L	1	1.6	-	-		-	-	-
**Total**	**64**	**100.0**		**46**	**100.0**		**16**	**100.0**

n: number of mutations identified; NRTIs: nucleotide reverse transcriptase inhibitors; NNRTIs: non-nucleotide reverse transcriptase inhibitors; PIs: protease inhibitors.

Frequency of participants with HIV-1 with mutations encoding for resistance to either dual or multiclass ARVs was analyzed: Majority of the participants had mutations encoding for both NRTIs + NNRTIs 16(32%); this was followed by NRTs + NNRTs + PIs 4(8%) and NRTIs + PIs 1(2%) the least (Table 3).

**Table 3:** frequency of participants with HIV-1 with mutations encoding for resistance to single, dual, and multiclass ARVs

Classes of ARVs	Frequency of participant with HIV-1 drug-resistant mutations (%)
NNRTIs	9(18%)
NRTIs	2(4%)
NRTIs, NNRTIs	16 (32%)
NRTIs, NNRTIs, PIs	4(8%)
NRTIs, PIs	1(2%)
PIs	2(4%)
**Total**	**34(68%)**

HIV-1: Human Immunodeficiency virus 1; ARVs: Antiretroviral; NRTIs: nucleotide reverse transcriptase inhibitors; NNRTIs: non-nucleotide reverse transcriptase inhibitors; PIs: protease inhibitors

## Discussion

The study was designed to determine the prevalence of HIV-1 drug-resistant mutations among adults on first and second-line antiretroviral therapy in a resource-limited health facility in Busia County, Kenya. More HIV-1 infection was frequently observed in females than males confirming earlier studies [20, 21]. This is because the prevalence of HIV-1 for females is more than males in the county and in the country. Additional analyses demonstrated that the ratio of females to males was similar, giving a platform of gender comparison as it is reported with National AIDS and STI Control Program that slightly more females are infected with HIV-1 than males [2, 22].

The current study recorded a higher prevalence of HIV-1 drug-resistance compared to prevalence recorded in the previous studies [6, 7, 15, 22], by a bigger margin. This implies that the problem of the drug-resistance will continue to rise until additional measures are put in the place. The world health organization guidelines that all persons infected with HIV-1 to be started on ARVs treatment irrespective of CD4 tally [8] might have contributed to this higher prevalence compared to prevalence in previous studies before this recommendation. Besides, the ART coverage (95%) in Busia county which is higher than the national (75%) [22] might have also contributed to this raised prevalence of HIV-1 drug-resistance. Further analysis showed no statistical significance difference between patients´ samples with mutations conferring resistance to first-line and second-line antiretroviral treatment with p >05. These findings in our study suggest resistance to second-line ARVs may rise soon thus posing a threat to the entire ARVs treatment.

The study identified major mutations encoding resistance to all the three classes of ARVs that are available in the country. The largest number of samples had mutations conferring resistance to NNRTIs and NRTIs then PIs the least. This results could be because NNRTIs and NRTIs which form the first-line ARVs have been in existence longer than PIs which form the second-line ART treatment drugs [23]. This observation is similar to the previous study which showed more HIV-1 infected Kenyan individuals are on NVP with a substantial percentage as high as 35% failing treatment and demonstrating poor adherence [24].

Our findings reveal major mutations conferring resistance to NRTIs with M184V, Y115F, K65R, D67N, K70E being prevalent. Major mutations conferring resistance to NNRTIs with K103N, Y181C, H22Y, G190A, K101E being prevalent have also been identified. PI major mutations were identified with I54V, F53L, V82A being prevalent. Some of these mutations have been identified in other similar studies in Kenya with K103N and M184V being predominant [7, 11, 15, 25, 26]. A scenario observed in the current study. However, the frequency of the multiple and specific mutations reported in this study is higher compared to the previous studies. Furthermore, 126 mutations identified in only 50 samples in this study is alarming in that these may put pressure on the existing ARVs not to work effectively. Further analysis of results revealed that many samples had resistant mutations to both NRTIs and NNRTIs, this could be the fact that the two classes of ARVs are the first-line ARVs and also many HIV-1 infected patients are started on these classes of ARVs before they are switched to second-line ARVs that consist of PIs [8]. A case seen in our study where many patients were taking first-line classes of drugs.

In this study, participants were taking seven different regimens of ARVs that are available. Further analysis showed that many participants were taking AZT+3TC+NVP; AZT+3TC+EFV and TDF+3TC+EFV as first-line HIV-1 treatment. This is because the former two drugs are ideal first-line ART for general population and the later drug is the substitute first-line ART regimen [27]. These outcomes conquer with the fact that many participants were put on these three drugs than other regimens of ARVs. Much as TDF+3TC+DTG was being taken up by very few participants simply because it is the latest recommended first-line drug of choice for use in Kenya [28, 29]. Multiple mutations identified in an individual taking this drug regimen pose a threat to entire first-line drugs. AZT+3TC+ATV/r was being taken by many participants compared to other second-line drugs perhaps due to the guidelines that after failure on the above ideal and substitute of first-line drugs, the patient be switched to AZT+3TC+ATV/r as a second-line drug [8, 28] of which is evidenced by our study that more patients were on the three first-line drugs compared to the rest. Although all samples had a viral load of more than 1000 copies per microliter of blood, most of them had resulted due to mutations as evidenced by our findings. However, a few samples without mutations could be due to other factors contributing to virologic failure [30].

Our study focused on the determining of HIV-1 drug-resistance by sequencing the pol region only. This would underestimate the overall prevalence of mutations. In that, some drugs target the envelope and gag regions of HIV as well. Therefore, we would recommend in such circumstances, all the three regions of the virus need to be sequenced.

## Conclusion

Multiple and multiclass mutations identified in the samples in this study poses a threat and serves as preliminary data in the initiation of the revising of the drug regimen combination. Furthermore, the study demonstrates a high prevalence of HIV-1 drug-resistance which calls for the strengthening of health programs especially in a resource-limited setting.

### What is known about this topic

- HIV-1 drug-resistance is a public health problem;
- Currently, all individuals infected with HIV-1 are started on treatment regardless of their CD4 tally;
- ART coverages are increasing in all the counties in Kenya.

### What this study adds

- Information in this study report existence of multiple and multi-class mutations in samples of the same individuals which is a threat to the treatment options;
- Evidence collected in this study supplements knowledge to the existing literature on HIV-1 drug-resistance example new mutations that were not identified before.
